# Prevalence and Pattern of Neurocognitive Impairment in Nigerians with Stages 3 to 5 Chronic Kidney Disease

**DOI:** 10.1155/2013/374890

**Published:** 2013-06-20

**Authors:** U. E. Williams, M. O. Owolabi, A. Ogunniyi, E. O. Ezunu

**Affiliations:** ^1^Internal Medicine Department, University of Calabar Teaching Hospital, Calabar 540242, Nigeria; ^2^Medicine Department, University College Hospital, Ibadan, Oyo State 200001, Nigeria; ^3^Federal Medical Centre, Asaba, Delta State 320241, Nigeria

## Abstract

*Background*. Cognitive impairment with its negative effect on quality of life has been reported in chronic kidney disease (CKD). The paucity of the literature on cognitive impairment in Africans with CKD prompted this study. *Objectives*. To determine the frequency and pattern of cognitive impairment in patients with stages 3 to 5 CKD. *Methods*. We studied 79 consecutive consenting adults with a National Kidney Foundation (NKF) stage 3 to 5 CKD based on their estimated glomerular filtration rate using the Cockcroft-Gault formula. The controls consisted of healthy demographically matched subjects. Community screening instrument for dementia (CSI'D), trail making test A (TMTA), and trail making test B (TMTB) were used for cognitive assessment. *Results*. More CKD patients had cognitive impairment compared with controls using CSI'D (51.9% versus 2.5%, *P* < 0.001); TMTA (53.2% versus 0%, *P* < 0.001); and TMTB (40% versus 0%, *P* < 0.001). The odds of having cognitive impairment increased in the presence of CKD when assessed using CSI'D (OR = 2.026; CI = 1.607–2.555); TMTA (OR = 3.13; CI = 2.40–4.09) and TMTB (OR = 3.22; CI = 2.42–4.25). CKD patients performed poorer on tests of executive function TMTA (*P* < 0.001) and TMTB (*P* < 0.001) while CSI'D showed significantly lower scores on multiple cognitive domains. *Conclusions*. Significant cognitive impairment in multiple domains exists among Nigerians with CKD.

## 1. Introduction

Chronic kidney disease (CKD) is a growing public health problem [1–4] with evidence pointing to an increasing incidence and prevalence worldwide [5, 6]. However, it is grossly underdiagnosed in Nigeria and other developing countries. The hospital frequency in Nigeria ranges between 1.6 and 8% of hospital admissions [7]. Although CKD affects all races equally, end-stage renal disease (ESRD) is four times more prevalent in blacks than whites in the United States [1]. 

CKD is found in persons of all ages, but higher incidence rates occur in patients at or above 65 years of age [8–10], an age range that is traditionally associated with a high prevalence of cognitive impairment. However, a lower average age has been reported among Nigerians with CKD [7]. Although data on prevalence and pattern of cognitive impairment among Nigerians with CKD is scanty, in other climes a high incidence of cognitive impairment has been reported among patients with CKD [1, 11–13]. Murray et al. [14] observed in their study of 338 haemodialysis patients that 37.0% had severe cognitive impairment which was more than three times the 5–10% estimated prevalence of dementia in the US population-based studies. Sehgal et al. [15] reported a prevalence of 22% of mild mental impairment among haemodialysis patients.

Cognitive impairment has been defined as a deficit in at least two aspects of cognitive function [1]. The key areas of cognition are attention, memory, language, visuospatial skills, and frontal/executive functions [14, 16]. When activities of daily living are not affected, cognitive impairment is said to be mild. Dementia, on the other hand, affects activities of daily living and behavior [1, 17]. There are no clearly stated guidelines on the ideal instrument or frequency of assessing cognitive impairment in patients with chronic kidney disease (CKD) [17]. Hence, several test instruments have been used in assessing cognitive impairment in CKD [1, 11, 18].

Furthermore, the lack of proper understanding of the pattern and risk factors for cognitive impairment has greatly affected the availability of treatment for this group of patients [1]. Therefore, cognitive impairment in CKD worsens the prognosis in patients [1, 19] and also increases the burden on medical and nonmedical care givers. It may hinder adherence to the complex regimens often prescribed to CKD patients, increase the risk of adverse events, and impair informed decision making [18]. It is associated with an increased number of hospital days and greater staff time after the termination of a dialysis session [20]. Moreover, cognitive impairment in CKD is independently associated with an increased mortality [20, 21] and dialysis withdrawal.

To mitigate these undesirable consequences more studies of cognitive dysfunction in CKD patients are required, particularly in Africa where few and inadequate reports are available. We aimed to evaluate the prevalence and pattern of cognitive impairment among Nigerians with stages 3 to 5 CKD in comparison with demographically matched controls while classifying CKD according to gold standard methods for estimating GFR.

## 2. Materials and Methods

### 2.1. Subjects

The study was a case-control study involving CKD patients at the University College Hospital (UCH), Ibadan, Nigeria. We included 79 consecutive adult patients aged 15 years and above seen in the medical wards, medical outpatient clinic, and dialysis unit of UCH with a National Kidney Foundation (NKF) [12] stage 3 to 5 CKD based on their estimated glomerular filtration rate (GFR) using the Cockcroft-Gault equation [22, 23]. Patients with a history of cerebrovascular disease, other neurological or psychiatric disorders, visual or auditory impairment, or delirium were excluded. The controls consisted of apparently healthy subjects matched for age, gender, and educational status, chosen from the hospital environment including patient relatives, medical and paramedical staff, with no history or laboratory parameters suggestive of CKD.

Ethical certification was obtained from the Research and Ethical Committee of the University College Hospital, Ibadan, Nigeria. Written informed consent was obtained from all the subjects. Participants were assured of confidentiality regarding all information given by them including their biodemographic data and other features of their illness.

Demographic data including age, sex, and level of education were obtained using a self-administered questionnaire. Clinical information including dry weight, duration of illness, and clinical features was obtained from their medical records. A general physical and detailed neurological examination was carried out on all subjects. Blood samples were taken within two days of assessment of cognitive function.

### 2.2. Assessment of Cognitive Function

Cognitive function was assessed using some items of the Community Screening Interview for Dementia (CSI'D) and the Trail Making Tests A and B (TMTA and TMTB). The CSI'D was developed by the Indianapolis-Ibadan Dementia Project group. It was validated at the University College Hospital, Ibadan, and in communities for the Ibadan-Indianapolis Dementia Project. Satisfactory validity and reliability have been reported in several populations with a sensitivity of 87% and specificity of 83% [24]. The CSI'D was designed specifically for use in non-literate and literate populations. The adaptation of its items to local language and culture makes it one of the most suitable instruments in our African setting [24].

The Trail Making Test A (TMTA) and Trail Making Test B (TMTB) are well-established tests sensitive to impairment in multiple cognitive domains [25–27]. Cognitive domains tested by the TMTA and TMTB include attention, concentration, visual scanning, psychomotor speed, and sequencing [28–30].

The selected items of the CSI'D and the Trail Making Tests were validated for the diagnosis of cognitive impairment in a subsample of normal subjects and normative values and cut-off scores were obtained. For the CSI'D, a score 2 standard deviation below the mean scores obtained from the pretest population was regarded as cognitively impaired. The cut-off points for the TMTA and TMTB in keeping with predetermined values were 90 and 180 seconds, respectively [26, 27].

In patients who had dialysis, the cognitive function was assessed at least twelve hours after the last dialysis session because the worst time to communicate with dialysis patients is usually during the dialysis session [17, 18].

### 2.3. Data Analysis

Numerical data were summarized as means and standard deviation, while categorical data were presented as frequencies and proportions. The difference in frequencies was analysed using chi-square test among the categorical variables, while the difference between means was tested using the Student's *t*-test. Significant level was set at *P* value less than 0.05. Data were analysed using the Statistical Package for the Social Sciences (SPSS) version 18.

## 3. Results

### 3.1. Characteristics of Study Subjects

Table 1 shows the demographic characteristics of the subjects. The male-to-female ratio was 1.9 : 1. There was no statistically significant difference in age between the patients (mean age ± SD) (39.7 ± 11.0 years) and controls (39.0 ± 11.9 years). All the CKD patients had at least primary school education. Although there was a slight difference in the educational levels attained by the controls, it was not statistically significant (Table 1). The greater proportion of the patients 42 (53.2%) had stage 5 CKD, while 28 (35.4%) were in stage 4 and 9 (11.4%) were in stage 3.

### 3.2. Frequency of Cognitive Impairment

**Figure 1:**
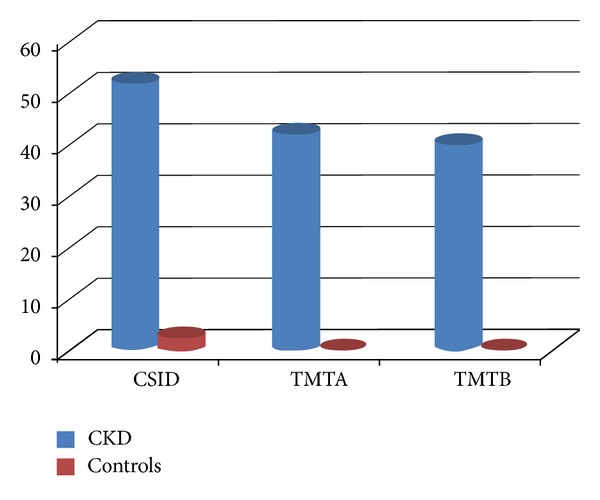
Frequency (%) of cognitive impairment among CKD patients and controls using CSID, TMTA and, TMTB.

More CKD patients (51.9%) had cognitive impairment compared with controls (2.5%), using CSI'D (chi-square test = *P* < 0.001Figure 1, Table 2). The odds of having cognitive impairment increased in the presence of CKD when assessed using CSI'D (OR = 2.026; CI = 1.607–2.555); TMTA (OR = 3.13; CI = 2.40–4.09), and TMTB (OR = 3.22; CI = 2.42–4.25, Table 2).

### 3.3. Pattern of CSI'D Performance in CKD Patients Compared to Controls

The mean CSI'D score was significantly lower among CKD patients compared to controls (Table 3). All the cognitive domains assessed by the elements of CSI'D recorded lower scores for the CKD patients compared to controls (Table 4). However, statistically significant lower scores were only obtained for language (naming) (*t* = 13.100, *P* < 0.001), language (fluency) (*t* = 9.760, *P* < 0.001), attention and calculation (*t* = 3.098, *P* < 0.001), orientation in place (*t* = 4.048, *P* < 0.001), immediate recall (*t* = 8.497, *P* < 0.001), and praxis (*t* = 10.504, *P* < 0.001).

### 3.4. Comparison of the Mean Time Taken to Complete TMTA and TMTB between Controls and CKD Patients

It took the CKD patients a mean of 102.6 ± seconds to complete the TMTA, which was much longer than the time taken by the controls (44.6 ± seconds, *P* < 0.001, Table 4). It took the CKD patients an average of 169.5 seconds to complete the TMTB compared to 78.3 second taken by the controls (*P* < 0.001).

## 4. Discussion

This cross-sectional study examined neurocognitive function using CSID, TMTA, and TMTB among patients with stages 3 to 5 CKD and compared the findings with those of demographically matched controls. We studied patients across all age ranges, with majority of the patients (31.6%) being between the ages of 41–50 years. This is similar to the age distribution of the patients studied by Ogunrin et al. [11, 31] and is therefore different from most studies carried out in Caucasian subjects which mostly assessed cognitive function in elderly patients with CKD [8, 13, 29].

The previous Nigerian study [32] had more female respondents with a female-to-male ratio of 1.3 : 1, but there were more males in this study at ratio of almost 2 : 1. Just like the study by Ogunrin et al. [31, 32] this study was based on consecutive renal patients in the hospital, while most of the studies in the western climes such as the INVADE study [2] were community-based studies. This is a pioneer study where cognitive function in patients with chronic kidney disease was assessed using the CSI'D. Other investigators had used TMTA [12], TMTB [8, 15], Iron Psychological Test Battery (FePsy) [32], the 6-item Cognitive Impairment Test (6CIT) [2], Modified Mini-Mental Scale Examination (3MS) [13, 14, 29], Mini Mental State Examination (MMSE), Event-Related Potential (ERP), and California Verbal Learning Trial (CVLT), among others [12].

A few studies evaluated mainly predialysis CKD patients [1, 33], while others enrolled CKD patients undergoing dialysis [22, 23]. This study, like that undertaken by Alebiosu and Ayodele [3] and Stivelman [28], included both predialysis and dialysed patients, but only about 25% of the patients had undergone dialysis. The proportion of CKD patients with cognitive impairment compared with that of controls from these studies is slightly different. Stivelman [28] comparing cognitive performance of eighty patients in stages 3 to 4 CKD with that of 80 patients in stage 5 CKD, observed that 38% of the patients with end-stage renal disease, 23% of the patients with advanced kidney disease, and 5% of the patients with mild-to-moderate CKD had cognitive impairment. The proportions of cognitive impairment among patients with moderate-to-severe CKD from our study on the different instruments were higher: 51.9% (CSI'D), 53.2% (TMTA), and 40.0% (TMTB). The higher proportion of cognitive impairment in our patients is due to the hospital-based nature of this study, the instruments used, and the fact that patients were mainly those in stages 3 to 5 chronic kidney disease of whom a greater proportion could not afford dialysis despite the fact that more than 50% of them had stage 5 CKD. 

The INVADE study [2], on the other hand, reported 9.9% among patients with mild CKD and 21.5% among patients with advanced CKD. Interestingly, however, they observed that 5.8% of the normal subjects had cognitive impairment, which was higher than the 2.5% observed among controls in this study. This slightly higher proportion might be due to the fact that the INVADE study considered mainly elderly community dwellers, unlike this study which had only 7.6% of the patients above 60 years. Advanced age is associated with most of the traditional risk factors for cognitive impairment and cerebrovascular diseases such as hypertension, diabetes mellitus, and dyslipidemia [2, 8, 34]. In another study, Murray et al. [14] classified 38% of the primary sample of 338 haemodialysis patients with cognitive impairment, which was also lower than the proportions observed in this study.

The CSI'D like MMSE and 3MS is a neuropsychological tool for assessing global cognitive function, while TMTA and TMTB predominantly evaluate attention, concentration, psychomotor speed, cognitive shifting, and complexing which are functions localised in the frontal lobe. It has been hypothesised that in the early stages of CKD, frontal lobe functions are selectively impaired, but at the later stages global cognitive function is impaired [8, 29, 35].

This study showed that in most cognitive domains assessed by the CSI'D including language (naming/fluency), attention and calculation, orientation (place/time), memory (immediate recall), and praxis, the CKD patients performed significantly poorer than the controls. Furthermore, the total CSI'D scores of CKD patients were lower than those of controls and the pretest population. This study further demonstrated statistically significant poorer performance on TMTA and TMTB in CKD patients than controls demonstrating the presence of impairment of frontal lobe executive functions in CKD patients with stages 3 to 5 CKD. One would conclude therefore that this study, in addition to demonstrating impairment in executive function, demonstrates that the CKD patients also had some impairment of global cognitive function.

Sehgal et al. [15] and Murray [33] had demonstrated that CKD patients performed poorly on visual/auditory reaction time task and verbal/nonverbal memory task compared to controls. With respect to concentration and attention, the renal failure patients in their study compared favourably with the controls on binary choice reaction task. Their findings regarding concentration and attention are not in keeping with our study and reports from other studies which showed global cognitive impairment among CKD patients [27]. Slinin et al. [29] and Stivelman [28] found an association between mild-to-moderate kidney disease and poor performance on TMTB, thus supporting our finding. Gelb et al. [36] observed that when cognitive functions in CKD patients were compared between controls and posttransplant renal patients, CKD patients had significantly worse verbal learning, verbal memory, and set-shifting task compared to other groups of subjects. This supports our finding of poorer performance in memory by the CKD patients.

## 5. Conclusions

This study shows that cognitive impairment is more frequent in Nigerian patients with stages 3 to 5 CKD patients than in demographically matched nonchronic kidney disease subjects. Chronic kidney disease is a risk factor for impairment of executive function, language, attention, calculation, orientation in place, immediate recall, and praxis. It is therefore recommended that cognitive function should be routinely assessed among patients with CKD.

## Figures and Tables

**Table 1:** Sociodemographic characteristics of subject.

Variable	Controls (*n* = 79)	CKD patients (*n* = 79)	Total (*n* = 158)	Test statistic	*P* value
Age group (years)					
11–20	2 (2.5)	2 (2.5)	4	Fisher's test	1.000
21–30	20 (30.5)	20 (30.5)	40		
31–40	19 (24.1)	19 (24.1)	38		
41–50	25 (31.6)	25 (31.6)	50		
51–60	7 (8.9)	7 (8.9)	14		
61–70	4 (5.6)	4 (5.6)	8		
≥71	2 (2.5)	2 (2.5)	4		
Sex					
Male	52 (65.8)	52 (65.8)	104	Chi-square test (0.0)	1.000
Female	27 (34.2)	27 (34.2)	54		
Education					
None	0 (0.0)	0 (0.0)	0	Chi-square test (0.0)	1.000
Primary	20 (25.3)	19 (24.1)	39		
Secondary	24 (30.4)	25 (31.6)	49		
Tertiary	35 (44.3)	35 (44.3)	70		

**Table 2:** Differences in frequency of cognitive impairment among CKD patients and controls.

	Cases *n* = 79 (% with cognitive impairment)	Controls *n* = 79 (% with cognitive impairment)	*χ* ^2^ value	*P* value	Odds ratio	95% CI
CSID	41 (51.9)	2 (2.5)	48.59	<0.001	2.03	1.61–2.56
TMTA	42 (53.2)	0 (0.0)	57.21	<0.001	3.13	2.40–4.09
TMTB	22 (40.0)^*♣*^	0 (0.0)	35.26	<0.001	3.22	2.42–4.25

^**♣**^Only 55 patients were able to complete TMTB on account of educational status.

**Table 3:** Comparison of the mean CSI'D performance between controls and CKD patients.

Variable	Mean (SD)	*t*-value	*P* value
Total CSI'D			
Control CKD	54.0 (5.0) 42.6 (5.9)	13.10	<0.001*
Language (naming)			
Controls CKD	6.9 (0.4) 5.7 (0.8)	11.78	<0.001*
Language (definition)			
Controls CKD	4.0 (0.1) 3.9 (0.4)	1.73	0.164
Language (fluency)			
Control CKD	15.6 (4.2) 9.4 (3.8)	9.76	<0.001*
Attention and calculation			
Controls CKD	3.0 (0.1) 2.8 (0.6)	3.10	<0.001*
Orientation (time)			
Controls CKD	4.9 (0.3) 4.6 (1.1)	2.26	0.019*
Orientation (place)			
Controls CKD	4.9 (0.5) 4.5 (0.8)	4.05	<0.001*
Memory (immediate recall)			
Controls CKD	4.6 (0.5) 4.5 (1.3)	8.50	<0.001*
Memory (registration)			
Controls CKD	3.1 (0.4) 3.0 (0.3)	0.47	0.256
Praxis			
Controls CKD	6.9 (0.4) 5.3 (1.3)	10.50	<0.001*

*Statistically significant.

**Table 4:** Comparison of the mean time taken to complete TMTA and TMTB among the controls and CKD patients.

Variable	Mean time in seconds (SD)	*t*-value	*P* value
TMTA			
Controls CKD patients	44.6 (16.7) 102.6 (43.6)	67.848	<0.001
TMTB			
Controls CKD patients	78.3 (28.9) 169.5 (56.0)	27.20	<0.001

## References

[1] Madero M, Gul A, Sarnak MJ (2008). Cognitive function in chronic kidney disease. *Seminars in Dialysis*.

[2] Levey AS, Atkins R, Coresh J (2007). Chronic kidney disease as a global public health problem: approaches and initiatives—a position statement from Kidney Disease Improving Global Outcomes. *Kidney International*.

[3] Alebiosu CO, Ayodele OE (2005). The global burden of chronic kidney disease and the way forward. *Ethnicity and Disease*.

[4] Afolabi MO, Abioye-Kuteyi EA, Arogundade FA, Bello IS (2009). Prevalence of chronic kidney disease in a Nigerian family practice population. *South African Family Practice*.

[5] (2002). NKF, K/DOQI clinical practice guidelines for chronic kidney disease: evaluation, classification, and stratification. *American Journal of Kidney Diseases*.

[6] Orth SR, Hallan SI (2008). Smoking: a risk factor for progression of chronic kidney disease and for cardiovascular morbidity and mortality in renal patients—absence of evidence or evidence of absence?. *Clinical Journal of the American Society of Nephrology*.

[7] Akinsola W, Odesanmi WO, Ogunniyi JO, Ladipo GOA (1989). Diseases causing chronic renal failure in Nigerians—a prospective study of 100 cases. *African Journal of Medicine and Medical Sciences*.

[8] Kurella M, Chertow GM, Luan J, Yaffe K (2004). Cognitive impairment in chronic kidney disease. *Journal of the American Geriatrics Society*.

[9] Brown EA (2008). Should older patients be offered peritoneal dialysis?. *Peritoneal Dialysis International*.

[10] Heras M, Fernández-Reyes MJ, Sánchez R (2010). Outcome implications of chronic kidney disease in the elderly. *Nefrologia*.

[11] Ogunrin AO, Unuigbe EI, Azubuike C (2006). Memory and perceptuo-motor performance in Nigerians with chronic renal impairment. *Medical Science Monitor*.

[12] Madan P, Kalra OP, Agarwal S, Tandon OP (2007). Cognitive impairment in chronic kidney disease. *Nephrology Dialysis Transplantation*.

[13] Kurella M, Chertow GM, Luan J, Yaffe K (2004). Cognitive impairment in chronic kidney disease. *Journal of the American Geriatrics Society*.

[14] Murray AM, Tupper DE, Knopman DS (2006). Cognitive impairment in hemodialysis patients is common. *Neurology*.

[15] Sehgal AR, Grey SF, DeOreo PB, Whitehouse PJ (1997). Prevalence, recognition, and implications of mental impairment among hemodialysis patients. *American Journal of Kidney Diseases*.

[16] Grabowski TJ, Anderson SW, Cooper GE (2002). Neural substrate of cognition. *Continuum: Lifelong Learning Neurol*.

[17] Gauthier S, Reisberg B, Zaudig M (2006). Mild cognitive impairment. *The Lancet*.

[18] Tamura MK, Yaffe K (2011). Dementia and cognitive impairment in ESRD: diagnostic and therapeutic strategies. *Kidney International*.

[19] Murray AM, Knopman DS (2010). Cognitive impairment in CKD: no longer an occult burden. *American Journal of Kidney Diseases*.

[20] Kurella M, Mapes DL, Port FK, Chertow GM (2006). Correlates and outcomes of dementia among dialysis patients: the dialysis outcomes and practice patterns study. *Nephrology Dialysis Transplantation*.

[21] Gussekloo J, Westendorp RGJ, Remarque EJ, Lagaay AM, Heeren TJ, Knook DL (1997). Impact of mild cognitive impairment on survival in very elderly people: cohort study. *British Medical Journal*.

[22] Melloni C, Peterson ED, Chen AY (2008). Cockcroft-Gault versus modification of diet in renal disease: importance of glomerular filtration rate formula for classification of chronic kidney disease in patients with non-ST-segment elevation acute coronary syndromes. *Journal of the American College of Cardiology*.

[23] Teruel Briones JL, Sabater J, Galeano C (2007). The Cockcroft-Gault equation is better than MDRD equation to estimate the glomerular filtration rate in patients with advanced chronic renal failure. *Nefrologia*.

[24] Hendrie HC, Ogunniyi A, Hall KS (2001). Incidence of Dementia and Alzheimer disease in 2 communities: yoruba residing in Ibadan, Nigeria, and African Americans residing in Indianapolis, Indiana. *Journal of the American Medical Association*.

[25] Jones BN, Teng EL, Folstein MF, Harrison KS (1993). A new bedside test of cognition for patients with HIV infection. *Annals of Internal Medicine*.

[26] Corrigan JD, Hinkeldey NS (1987). Relationships between parts A and B of the Trail Making Test. *Journal of Clinical Psychology*.

[27] Oliveira-Souza RD, Moll J, Passman LJ (2000). Trail marking and cognitive-shifting. *Arquivos de Neuro-Psiquiatria*.

[28] Stivelman JC (2000). Benefits of anaemia treatment on cognitive function. *Nephrology Dialysis Transplantation*.

[29] Slinin Y, Paudel ML, Ishani A (2008). Kidney function and cognitive performance and decline in older men. *Journal of the American Geriatrics Society*.

[30] Gaudino EA, Geisler MW, Squires NK (1995). Construct validity in the trail making test: what makes Part B harder?. *Journal of Clinical and Experimental Neuropsychology*.

[31] Uniegbe E, Ogunrin O, Onyemekeihia R (2004). Neurocognitive performances in Nigerian Africans with chronic renal failure. *Nigerian Journal of Health and Biomedical Sciences*.

[32] Ogunrin AO, Unuigbe EI, Azubuike C (2006). Memory and perceptuo-motor performance in Nigerians with chronic renal impairment. *Medical Science Monitor*.

[33] Murray AM (2008). Cognitive impairment in the aging dialysis and chronic kidney disease populations: an occult burden. *Advances in Chronic Kidney Disease*.

[34] Hailpern SM, Melamed ML, Cohen HW, Hostetter TH (2007). Moderate chronic kidney disease and cognitive function in adults 20 to 59 years of age: third National Health and Nutrition Examination Survey (NHANES III). *Journal of the American Society of Nephrology*.

[35] Jassal SV, Roscoe J, LeBlanc D, Devins GM, Rourke S (2008). Differential impairment of psychomotor efficiency and processing speed in patients with chronic kidney disease. *International Urology and Nephrology*.

[36] Gelb S, Shapiro RJ, Hill A, Thornton WL (2008). Cognitive outcome following kidney transplantation. *Nephrology Dialysis Transplantation*.

